# Effects of Stimulants of the Voice

**Published:** 1889-11

**Authors:** Morrell Mackenzie


### EFFECT OF STIMULANTS ON THE VOICE.

Tobacco, alcohol, and fiery condiments of all kinds are best avoided by those who have to speak much, or, at least, they should be used in strict moderation. I feel bound to warn speakers addicted to the "herb nicotian" against cigarettes. Like tippling, the effect of cigarette smoking is cumulative, and the slight but constant absorption of tobacco juice and smoke makes the practice far more noxious in the long run than any other form of smoking. Our forefathers, who used regularly to end their evenings under the table, seemed to have suffered little of the well known effects of alcohol on the nerves, while the modern tippler, who is never intoxicated, is a being whose whole nervous system may be said to be in a state of chronic inflammation. In like manner cigarette smokers (those at least who inhale the smoke, and do not merely puff it "from the lips outward," as Carlyle would say), are often in a state narcotic poisoning. The old jest about the slowness of the poison may seem applicable here, but though the process may be slow there can be little doubt that it is sure. Even if it does not kill the body, it too often kills or greatly impairs the victim's working efficiency and usefullness in life. The local effects of cigarettes in the mouth must also be taken into account by those whose work lies in the direction of public speech. The white spot on the tongue and insides of the cheeks, known as "smoker's patch," are believed by some doctors with special experience to be more common in devotees of the cigarette than in other smokers ; this unhealthy condition of the mouth may not only make speaking troublesome, or even painful, but it is now proved to be a predisposing cause of cancer. All fiery or pungent foods, condiments, or drinks tend to cause congestion of the throat, and if this condition becomes chronic it may lead to impairment, if not complete loss, of the voice. The supposed miraculous virtues of the mysterious possets and draughts on which some orators pin their faith exists mainly in the imagination of those who use them ; at beat they do nothing more than lubricate the joints of the vocal machine so as to make it work more smoothly.—*Sir Morrell Mackenzie in the Contemporary Review*

